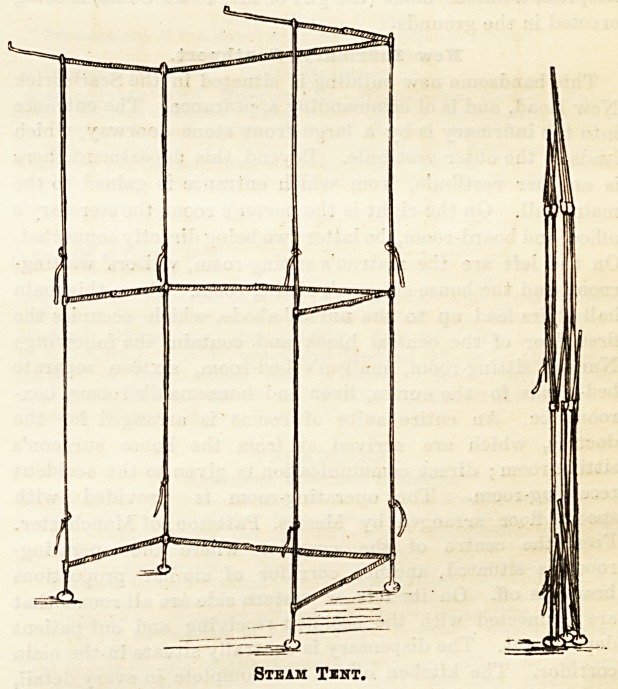# Practical Departments

**Published:** 1895-04-27

**Authors:** 


### PRACTICAL DEPARTMENTS.

### APPLIANCES AT KING'S COLLEGE HOSPITAL.

A walk through the wards of King's College Hospital cannot fail to be of particular interest to all concerned with the internal economy of hospital life. Perhaps in no other hospital in London are to be found such evidences of individuality as at King's College; at every turn the observant visitor finds traces of the thoughtful care which has been expended upon every detail, no matter how apparently trivial, of the ward fittings and appliances.

Ophthalmic Dressing Tray.

Miss Monk, the "Sister Matron" is gifted with inventive powers to no small degree, and the result is seen in numberless ways throughout the building. By permission, we give a few sketches of some of the more notable instances in which Miss Monk's ideas have been carried out in her wards to the manifest advantage of all concerned.

Tracheotomy Box.

Exceedingly complete dressing-trays are in use in the ophthalmic wards, holding everything needed by doctor and nurse, and light enough to be carried easily from bed to bed as required. They are made of black japanned tin, in four divisions, containing respectively wool, pads and bandages, lotions and ointments. The lid inverted is used to hold the porringers for lotions, &c., as will be seen in the first illustration.

The second sketch is of a tracheotomy box, which contains all requisites for the operation. In the lower half are kept gauze, feathers, macintosh, ligatures, tape, &c., while the top tray is divided into three, where are kept forceps, scalpels, tubes, &c. In the lid are places for cards, giving a list of every article which should be found in the box. Both these and the ophthalmic dressing-tray are of japanned tin, and are made by Messrs. Hepburn and Cox, deed box manufacturers.

Stand for Holding Syringes.

The third drawing represents a very excellent arrangement for keeping and preserving syringes in good order. Each stand is made to hold four syringes suspended with the nozzle over a small tray, which fits into the space at the bottom and can be easily removed for cleaning purposes. Each ward is provided with one of these stands hanging against the wall in its appointed corner. They are also of japanned tin.

Steam Tent.

The frame for supporting a steam tent, which is shown below, is really quite a marvel of ingenuity and convenience, folding up into a comparatively small compass for stowing away when not in use. There is a small omission in the sketch here given, which does not give the front rod connecting the side pieces at the top upon which the tent rests.

These and many other similar contrivances have been made under Miss Monk's special supervision, and are excellent in every way. We should not forget to mention the beautiful operating table in the theatre, the joint invention of the matron and one of the surgeons of King's College, and presented by them to the hospital. Of this we shall hope to give an illustration on another occasion.

## Figures and Tables

**Figure f1:**
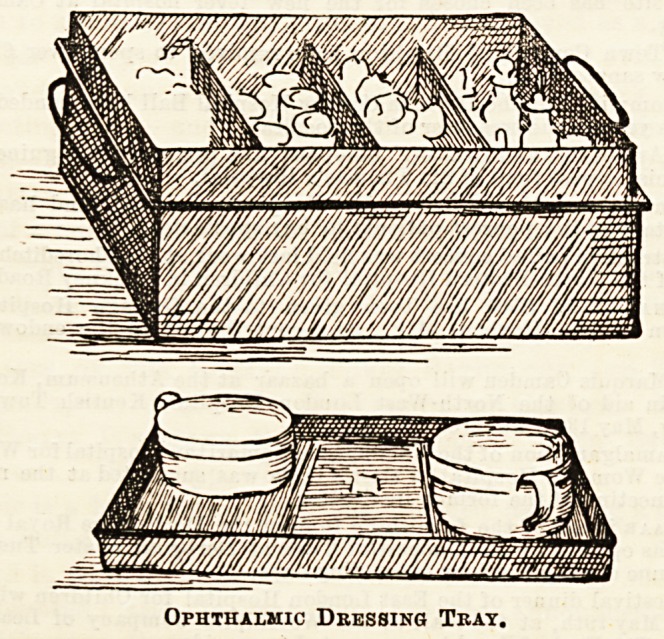


**Figure f2:**
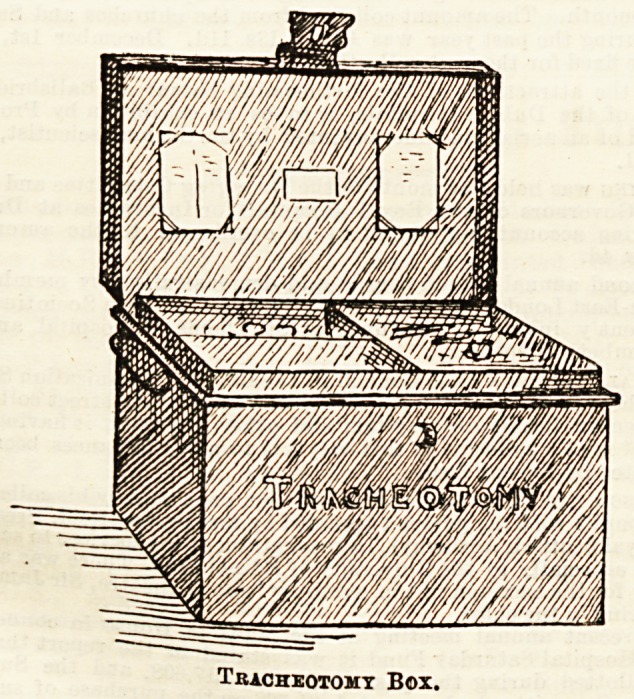


**Figure f3:**
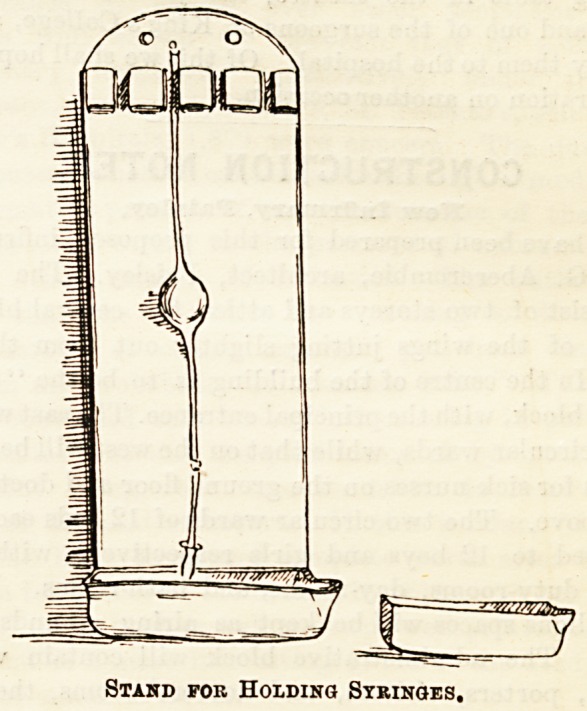


**Figure f4:**